# Neuroprotective epi-drugs quench the inflammatory response and microglial/macrophage activation in a mouse model of permanent brain ischemia

**DOI:** 10.1186/s12974-020-02028-4

**Published:** 2020-11-27

**Authors:** Mariana Mota, Vanessa Porrini, Edoardo Parrella, Marina Benarese, Arianna Bellucci, Sina Rhein, Markus Schwaninger, Marina Pizzi

**Affiliations:** 1 Division of Pharmacology, Department of Molecular and Translational Medicine, University of Brescia, Viale Europa 11, 25123 Brescia, Italy; 2 Institute for Experimental and Clinical Pharmacology and Toxicology, University of Lübeck, Lübeck, Germany

**Keywords:** Stroke, Inflammation, Microglia, NF-kB RelA, Resveratrol, MS-275

## Abstract

**Background:**

Activation of NF-kappaB RelA deacetylated at the lysine residues, except the lysine 310, drives pro-apoptotic transcription in noxious brain ischemia. We showed that the sinergistic combination of the histone deacetilase inhibitor MS-275 with the sirtuin 1 activator resveratrol, at very low doses, restores normal RelA acetylation and elicit neuroprotection in mice subjected to transient middle cerebral artery occlusion (tMCAO) and primary cortical neurons exposed to oxygen-glucose-deprivation (OGD). The present study aims at corroborating the neuroprotective potential of the epigenetic treatment in a model of permanent brain ischemia and investigate its effect on post-ischemic inflammation and microglia activation.

**Methods:**

Male mice subjected to permanent occlusion of the distal MCAO (pMCAO) were treated with vehicle or MS-275 (20 μg/kg) and resveratrol (680 μg/kg) i.p. immediately after the ischemia. Microglia-containing mixed glial cultures were prepared from the brain of 1–3-day-old mice. Primary cortical neurons were prepared from 15-day-old embryonic mice.

**Results:**

MS-275 and resveratrol in combination, but not individually, reduced infarct volume and neurological deficits evaluated 48 h after the pMCAO. At 24 h, the treatment inhibited the RelA binding to *Nos2* promoter, reduced the elevated expression of *Nos2, Il6, Il1b, Mrc1* and *Ym1* and the leukocytes infiltration in the ischemic area. The effect was nonpermanent. The treatment did not limit the sustained leukocyte infiltration or *Nos2* and *Il1b* transcription observed at 7 days. Though, it induced alternative activation markers of microglia/macrophages, *Arg1, Ym1* and *Fcgr2b* that could be added to *Mrc1, Tgfb1* and *Trem2* spontaneously increased at 7 days after ischemia.

At 24 hours the drug treatment quenched the microglia/macrophages activation in the ischemic cortical sections, as shown by the recovered ramified morphology and lowered iNOS or CD68 immunoreactivity in Iba1-positive cells.

Both microglia and astrocytes in mixed glial cultures, but not pure astrocytes, displayed signs of activation and iNOS-immunoreactivity when treated with a conditioned medium (NCM) from OGD-exposed cortical neurons. The epigenetic drugs limited the OGD-NCM-mediated activation.

**Conclusions:**

Our findings indicate that single treatment with MS-275 and resveratrol can reduce stroke-mediated brain injury and inflammation observed 2 days after the pMCAO and put the rational to test repeated administration of the drugs. The anti-inflammatory property of MS-275 and resveratrol combination can be ascribed to both primary direct inhibition of microglia/macrophage activation and secondary glial/macrophages inhibition mediated by neuroprotection.

**Supplementary Information:**

The online version contains supplementary material available at 10.1186/s12974-020-02028-4.

## Background

Acute ischemic stroke is a leading cause of death and disability worldwide [1, 2]. This disease is characterized by damage to the brain tissue surrounding the occluded cerebral artery, most commonly the middle cerebral artery [3]. Treatment options are currently very limited, and the only available pharmacological treatment, recombinant tissue plasminogen activator (rtPA), has a very short 4.5-hour time window, which benefits only about 5% of all stroke patients [4]. As such, there is a need for the development of new therapies that reduce post-ischemic neuronal injury.

The ischemic insult triggers a series of pathological processes, including excitotoxicity, apoptosis and inflammation, which contribute to cell death [5].

The nuclear factor-kappa B (NF-κB) family of transcription factors has diverse functions in the CNS. Under physiological conditions, NF-κB is a key regulator of cell survival, development, synaptic plasticity [6], and long-term memory [7]. By being a master regulator of the inflammatory and apoptotic processes, this transcription factor has also been associated with both acute and chronic neurodegenerative diseases, including, but not limited to, stroke, Alzheimer’s, Parkinson’s, and Huntington’s diseases [8, 9]. Five subunits compose the NF-κB family of proteins: RelA (p65), RelB, c-Rel, p50, and p52, found in the cytoplasm as homo- or heterodimers. The activation of NF-κB plays both neuroprotective and neurotoxic roles depending on the subunits forming the transcription factor.

In particular, p50/RelA dimers are activated by neurotoxic stimuli, such as ischemia [10, 11], glutamate [12], or β-amyloid [13]. This induces the transcription of pro-apoptotic factors, like Bim and Noxa [10], as well as the 1B isoform of the divalent metal transporter-1 (1B/DMT1), a membrane carrier responsible for iron accumulation and brain damage after injury [14].

We showed that p50/RelA exerts opposite effects depending on the acetylation state of RelA, and specifically on the acetylation on K310 [15, 16]. In fact, either neuroprotective preconditioning or lethal ischemia activate p50/RelA to the same extent. However, only in lethal oxygen and glucose deprivation (OGD) activated RelA shows a general deacetylation but a site-specific acetylation at the K310 residue, which favors pro-apoptotic transcription [15]. Acetylated RelA at K310 was found to interact with the CREB-binding protein (CBP) [16], detach from the anti-apoptotic *Bcl-χ*_*L*_ promoter and bind to the pro-apoptotic *Bim* promoter [15].

In recent years, a significant body of data has shown that post-ischemic treatment with either histone deacetylase inhibitors (HDACi) [17–21] or the sirtuin activator resveratrol [22–25], is neuroprotective in experimental models of brain ischemia. We recently demonstrated that the combination of class I HDACi MS-275 (entinostat) with resveratrol, at very low doses, is able to correct the abnormal acetylation state of RelA, lowering the ratio K310/total acetylation in models of transient brain ischemia and amyotrophic lateral sclerosis [15, 26]. The combination of epigenetic drugs also restored the acetylation of H3 histones affected by lethal ischemia [15]. The synergistic action resulted from resveratrol-stimulated AMP-activated kinase (AMPK), which sustained the lysine acetilation by histone acetyltransferases (HATs) activity, upon HDAC inhibition by MS-275, and resveratrol-stimulated SIRT1 which targeted RelA K310 acetylation [15]. Concomitantly, the treatment induced a transcriptional switch and RelA binding from the pro- apoptotic *Bim* gene to anti-apoptotic *Bcl-χ*_*L*_ gene. In mice subjected to transient middle cerebral artery occlusion (tMCAO), MS-275 and resveratrol were neuroprotective when administered up to 7 hours after the insult. The beneficial effect was still evident after 72 hours [15].

With the aim to add translational power to the preclinical findings, we studied the effect of the epigenetic drugs in a model of permanent ischemic stroke that in comparison with the common models of transient ischemic stroke exhibits higher neuroinflammation and leukocyte infiltration [27].

Permanent occlusion of the middle cerebral artery (pMCAO) in mice produces an injury with good reproducibility of infarct size and neurological deficits [28]. Moreover, by mimicking stroke without reperfusion, it models the majority of clinical stroke cases [29]. MS-275 and resveratrol were tested in mice subjected to pMCAO to evaluate their anti-inflammatory potential and their capability to reduce infarct volume and promote neurologic recovery. The drug combination was also tested in primary mixed glial cultures incubated with a conditioned medium from OGD-exposed cortical neurons.

We here show that even in the pMCAO model the epigenetic drug combination of MS-275 and resveratrol limited the post-ischemic brain damage. Moreover, this treatment reduced the neuroinflammatory response associated with microglia activation, by directly quencing microglial reactivity.

## Methods

### Drugs

MS-275 and resveratrol were purchased from Vinci Biochem (Vinci, Italy) and Merck Millipore (Burlington, MA, USA), respectively. The compounds were dissolved in dimethylsulfoxide (DMSO, Sigma-Aldrich, Saint Louis, MO, USA).

### Animals

Male C57BL/6NCrl mice (8- to 9-week old, 20-25 g, Charles River, Germany) were used. All experiments were performed according to the German animal protection law and were approved by the local animal welfare authorities (Ministerium für Energiewende, Landwirtschaft, Umwelt, Natur und Digitalisierung, Kiel, Germany). Before beginning any procedure, mice were housed for at least a week in ventilated cages in groups of four to five individuals in a 12/12 hour light/dark cycle at 23°C, with ad libitum access to food and water.

### pMCAO stroke model

To induce ischemic stroke, mice were subjected to permanent occlusion of the distal MCA [30]. Briefly, mice were anaesthetized with tribromoethanol (15 μL of 2.5% tribromoethanol/g body weight, i.p.) and an incision was made between the ear and the orbit on the left side. The temporal muscle was removed and a burr hole was drilled to expose the MCA, which was then occluded by bipolar electrocoagulation (Model ICC 50, Erbe, Marietta, GA, USA). The surgery was done under a microscope (Hund) and rectal temperature was maintained at 37 °C during surgery by a heating pad. After the incision was closed by a suture, mice were placed under a heating lamp until fully recovered. Animals undergoing sham surgery went through the same surgical procedures, but without occlusion of the MCA. Mice were randomized to the treatment groups. MS-275 and resveratrol were dissolved in saline with 1% (v/v) DMSO at the doses of 20 μg/kg and 680 μg/kg, respectively, and intraperitoneally administered immediately after the pMCAO procedure. Mortality was <15% and did not differ between treatment groups.

### Behaviour analysis

To evaluate sensorimotor function, two established tests were used: the corner and latency-to-move tests [30]. Briefly, in the corner test, mice were allowed to enter a 30x20 cm corner with an angle of 30° 24h before (considered as the baseline value) and 48h after pMCAO, and the number of right and left turns on rearing out of 12 trials were counted, or for a maximum of 30 minutes. For the latency-to-move test, mice were placed at the center of a plain board. The time to cross one body length (7cm) was measured three times for each mouse, 24h before and 48h after pMCAO.

### Infarct volume

Forty-eight hours after pMCAO, mice were deeply reanaesthetized with tribromoethanol and intracardially perfused with Ringer’s solution. Brains were removed and coronally cryosectioned (20-μm thick) every 400 μm. Coronal sections were stained with a silver technique as described previously [31]. Stained sections were scanned at 600 dpi and the infarct area was measured. The total infarct volume was obtained from integrating infarcted areas after subtracting the difference between the area of the ischemic and the non-ischemic hemispheres to correct for brain edema [30].

### Real-time quantitative reverse transcription-polymerase chain reaction (qRT-PCR)

Analysis of mRNA expression of inflammatory markers was performed 1 and 7 days day after pMCAO. Total RNA was purified from ipsilateral hemispheres using the RNeasy Mini Kit for total RNA extractions (Qiagen, Hilden, Germany), according to the manufacturer’s instructions. Then, 1 μg of total RNA was transcribed to cDNA using the Quantitect® Reverse Transcription Kit (Qiagen, Hilden, Germany) with optimized mix of oligo-dT and random primers as primer. The following primers were used for quantitative RT-PCR: *Arg1* forward GTGTACATTGGCTTGCGAGA, *Arg1* reverse AATCGGCCTTTTCTTCCTTC, *Fcgr3* forward TATCGGTGTCAAATGGAGCA, *Fcgr3* reverse GCACCTTAGCGTGATGGTTT, *Fcgr2b* forward AGGGCCTCCATCTGGACTG, *Fcgr2b* reverse GTGGTTCTGGTAATCATGCTCTG, *Gapdh* forward TCAACAGCAACTCCCACTCTT, *Gapdh* reverse CCAGGGTTTCTTACTCCTTGG, *Il6* forward CCTACCCCAATTTCCAATGCT, *Il6* reverse TATTTTCTGACCACAGTGAGGAAT, *Il1b* forward GGCTTCAGGCAGGCAGTATC, *Il1b* reverse TAATGGGAACGTCACACACC, *Nos2* forward AGCCAAGCCCTCACCTACTT, *Nos2* reverse GTGGGGTTGTTGCTGAACTT, *Mrc1* forward AAGGTTCGGGATTGTGGAG, *Mrc1* reverse TCGTAGTCAGTGGTGGTTCC, *Tgfb1* forward CAATTCCTGGCGTTACCTTG, *Tgfb1* reverse GGTTCATGTCATGGATGGTG, *Trem2* forward CACTCTGAAGAACCTCCAAGC, *Trem2* reverse ATTCCTGGAGGTGCTGTGTT, *Ym1* forward GCCCACCAGGAAAGTACACA, *Ym1* reverse CACGGCACCTCCTAAATTGT.

cDNA (1-5 μL) was amplified with iQ™ SYBR Green Supermix (Bio-Rad, Hercules, CA, USA) and forward and reverse primers (10 μM). Each reaction was performed in triplicate, using the fast program in ViiA 7 Real-Time PCR System (Applied Biosystems, Foster City, CA, USA). For standardization of quantification, *Gapdh* was used as housekeeping gene. Data were analyzed following the comparative Ct method, where dCt: Housekeeping Ct - Target Ct; and ddCt: Calibrator dCt - Sample dCt.

### Chromatin immunoprecipitation and real-time PCR analysis

Chromatin immunoprecipitation (ChIP) assays were performed to study RelA interactions and H3 histone acetylation at the *Nos2* and *Il6* promoters in ipsilateral hemispheres of mice one day after pMCAO, using a SimpleChIP® Enzymatic Chromatin IP Kit (Magnetic Beads) (#9003S, Cell Signaling Technology, Danvers, MA, USA) as previously described [15], with some modifications. Briefly, frozen brain tissue from the ipsilateral hemispheres of 2 animals per group were pooled and chopped into small pieces. Proteins were crosslinked to DNA using 1.5% formaldehyde (Sigma-Aldrich, St. Louis, MO, USA). The crosslinked chromatin was extracted using buffers A and B provided in the kit, digested by micrococcal nuclease, and sonicated on ice with an ultrasonic homogenizer (5 cicles, 20 sec, 50% power output, Bandelin SONOPLUS, Berlin, Germany). Chromatin was immunoprecipitated with antibodies anti-acetyl H3(K9/18) (Upstate-Millipore #07-593, Burlington, MA, USA), anti-RelA (Santa Cruz Biotechnology #sc-372X, Dallas, TX, USA) and anti-IgG (negative control), overnight at 4°C with gentle rotation. The complexes were captured by magnetic-coupled protein G beads for 2 h at 4 °C with gentle rotation. Following washing, bound DNA fragments were eluted and analyzed by subsequent real-time qRT-PCR using the following primers: *Nos2* forward CCACAGAGTGATGTAATCAAGCA, *Nos2* reverse GCAGCAGCCATCAGGTATTT, *Il6* forward CCCACCCTCCAACAAAGATT, *Il6* reverse TGAGCTACAGACATCCCCAGT. Immunoprecipitated DNA (3 μl) was amplified with iQ™ SYBR Green Supermix (Bio-Rad, Hercules, CA, USA) and forward and reverse primers (10 μM). Each reaction was performed in triplicate, using the fast program in ViiA 7 Real-Time PCR System (Applied Biosystems, Foster City, CA, USA). Ct values obtained by qRT-PCR analysis of samples immunoprecipitated with anti-RelA or anti-acetyl-H3 antibodies were normalized over corresponding Ct values obtained by IgG immunoprecipitation, and further normalized over relative Ct values obtained in INPUT (no immunoprecipitated) chromatin. Final data obtained in mice subjected to pMCAO or pMCAO plus treatment were normalized to the data obtained in sham operated mice.

### Immunohistochemistry

One or 7 days after pMCAO, mice were anaesthetized with tribromoethanol and perfused transcardially with Ringer’s solution and 4% paraformaldehyde (PFA) in phosphate buffered saline (PBS). Brains were removed and postfixed in 4% PFA at 4°C for 2h, followed by cryoprotection in 30% sucrose in PBS overnight (O/N) at 4°C. After snapfreezing in isopentane (Sigma-Aldrich, St. Louis, MO, USA) cooled in liquid nitrogen, 15-μm-thick coronal cryosections were mounted on Superfrost Plus slides and preserved at -80°C until use.

For double immunofluorescence staining, tissues were permeabilized with 0.3% Triton X-100 (Sigma-Aldrich, St. Louis, MO, USA) in 20% methanol in PBS for 30 min and then blocked with PBS containing 3% bovine serum albumine (BSA) (Sigma-Aldrich, St. Louis, MO, USA), 2% normal goat serum (Sigma-Aldrich, St. Louis, MO, USA) for 2 h at RT. Sections were incubated overnight at 4°C with the first primary antibody in blocking solution: mouse anti-iNOS (1:50, BD Biosciences #610329, Franklin Lakes, NJ, USA), rat anti-CD68 (1:100, Bio-Rad #MCA1957, Hercules, CA, USA), or rat anti-MRC1 (1:100, Bio-Rad #MCA2235, Hercules, CA, USA). The following day, slices were incubated with the secondary antibody for 1h at RT in PBS containing 1% BSA and 0.3% Triton X-100: for iNOS, biotinylated goat anti-mouse (1:600, Vector laboratories #BA-9200, Burlingame, CA, USA), or for CD68 and MRC1, biotinylated goat anti-rat (1:600, Vector laboratories #BA-9401, Burlingame, CA, USA) followed by streptavidin-Alexa Fluor 594 (1:1500, ThermoFisher Scientific #S11227, Waltham, MA, USA). Then, sections were incubated 2h at 37°C with the second primary antibody, rabbit anti-Iba1 (1:1000, Wako #019-19741, Osaka, Japan), in blocking solution. Slices were incubated in the second secondary antibody, goat anti-rabbit Alexa Fluor 488 (1:1500, Jackson ImmunoResearch #111-545- 144, West Grove, PA, USA), for 1h at RT in PBS containing 1% BSA and 0.3% Triton X-100. Finally, sections were incubated with Hoechst 33342 (1 mg/mL, Sigma-Aldrich, St. Louis, MO, USA) for 3 minutes to stain cellular nuclei and mounted with Vectashield (Vector Laboratories, Burlingame, CA, USA).

3,3’-Diaminobenzidine (DAB) immunostaining tissues were permeabilized with 0.3% Triton X-100 (Sigma-Aldrich, St. Louis, MO, USA) in 20% methanol in PBS for 20 min and then blocked with PBS containing 5% BSA (Sigma-Aldrich, St. Louis, MO, USA) for 2 h at RT. Slices were incubated overnight at 4°C with the primary antibody anti- CD45 (1:70 Bio-Rad #MCA1031, Hercules, CA, USA) in blocking solution. The following day, brain sections were incubated with secondary antibody, biotinylated goat anti-rat (1:500, Vector laboratories #BA-9401, Burlingame, CA, USA), for 1h at RT in PBS containing 1% BSA and 0.3% Triton X-100. Finally, the signal was visualized by avidin-biotin-horseradish peroxidase technique (ABC Elite; Vector Laboratories, Burlingame, CA, USA) using ImmPACT™ DAB kit (Vector Laboratories, Burlingame, CA, USA) as chromogen.

Quantification of CD45 immunoreactivity was performed on digitized images using ImageJ software [32]. Briefly, brains from 3 mice (3-4 sections from each mouse) were analyzed by examining an average of 4 fields per section. Data were assessed as CD45 positive pixels in the ipsilateral hemisphere/CD45 positive pixels in the contralateral hemisphere and indicated as CD45 staining percentage area (ipsilateral/contralateral).

### Primary cell cultures

Primary cell cultures were obtained from C57Bl/6 mice (Charles River, Calco, Italy). The animal studies were approved by the Animal-welfare body of the University of Brescia and were in accordance with the Directive 2010/63/EU on the protection of animals used for scientific purposes. In details, primary cultures of cortical neurons were prepared from 15-day-old embryonic mice and cultured as previously described [33]. The cells were seeded at the density of 2 × 10^6^ cells in 21 cm^2^ culture dishes (Nunc, Germany) using Neurobasal medium (Invitrogen Corporation, Carlsbad, CA, USA) supplemented with 2% B27 (Invitrogen Corporation, Carlsbad, CA, USA), 0.5 mM L-glutamine (Euroclone, Pero, Italy) and 50 U/mL penicillin/streptomycin (Euroclone, Pero, Italy). The neurons were used after 11 days in vitro (DIV). Mixed glial cell cultures were prepared from the brains of 1–3-day-old mice [34]. The cells were grown in 24-well-plates at the density of 1.2 × 10^5^ cells per well using DMEM medium supplemented with 10% fetal bovine serum (FBS) (Euroclone, Pero, Italy), 4 mM L-glutamine (Euroclone, Pero, Italy) and 200 U/ml penicillin/streptomycin (Euroclone, Pero, Italy). Mixed glial cells were used after 14 DIV. For immunocytochemistry (ICC) studies, a coverslip was included in the well. Approximately 10% of microglia was present in the mixed microglia-astrocyte cultures. Pure astrocytes were isolated by shaking the flasks containing the mixed glia cells for 6 hours [35]. They were seeded in 24-well-plates at the density 1.2 × 10^5^ cells per well and used after 14 DIV. For ICC experiments the cells were grown on coverslips.

### Oxygen and glucose deprivation

Primary cultures of mouse cortical neurons were exposed to OGD for 3h according to previous experiments [33]. Briefly, cells were incubated with deoxygenated glucose-free balanced salt solution and transferred to an air-tight chamber fluxed with 95% N_2_ and 5% CO_2_ for 10 min to reach an O_2_ concentration lower than 0.4%. A parallel set of cultured neurons were incubated for 3 h with normally oxygenated, balanced salt-solution containing glucose and used as a control. After the anoxic insult, cortical neurons were transferred to recover in neurobasal medium containing 0.4% B27 supplement.

### Preparation of neuron-derived conditioned medium and measurement of lactate dehydrogenase release

Neuron-derived Conditioned Medium (NCM) was prepared by diluting 1:5 in DMEM medium the medium collected from primary neuronal cultures subjected to 3 h OGD and 24 h recovery (NCM-OGD) or from neurons that underwent the same protocol except OGD (NCM-control). Primary mixed glia and pure astrocytes cultures were then incubated with NCM overnight. The duration of NCM incubation was chosen in order to detect cellular damage but no major cell death. Glial cell cultures were treated with MS-275 (0.1 μM) and resveratrol (3 μM), or vehicle, concomitantly with NCM incubation. At the end of NCM exposition period, we estimated the cellular injury by assessing the ratio between the amount of lactate dehydrogenase (LDH) released in culture medium and the total releasable LDH using the CytoTox 96® Non-Radioactive Cytotoxicity Assay (Promega, Fitchburg, WI, USA).

### Immunocytochemistry

Double immunofluorescence staining for Iba1 and GFAP was initiated by performing permeabilization and blocking, followed by an incubation at room temperature for 2 h with the primary antibody rabbit anti-Iba1 (1:900, Wako #019-19741, Osaka, Japan), and then by a 1-h incubation at room temperature with the secondary antibody goat anti-rabbit Alexa Fluor 488 (1:800, Jackson ImmunoResearch #111-545- 144, West Grove, PA, USA). Then, cells were incubated overnight at 4°C with the second primary antibody mouse anti-GFAP (1:200, Sigma-Aldrich #G3893 St. Louis, MO, USA) and then the second secondary antibody goat anti-mouse Cy3 (1:500, Jackson ImmunoResearch #115-165-003, West Grove, PA, USA) for 1 h at room temperature. Double immunofluorescence staining for Iba1 and iNOS was initiated by performing permeabilization and blocking, followed by an incubation at room temperature for 2 h with the primary antibody rabbit anti-Iba1 (1:900, Wako #019-19741, Osaka, Japan), and then by a 1-h incubation at room temperature with the secondary antibody goat anti-rabbit Alexa Fluor 488 (1:800, Jackson ImmunoResearch #111-545- 144, West Grove, PA, USA). Then, cells were incubated overnight at 4°C with the second primary antibody mouse anti-iNOS (1:25, BD Biosciences #610329, Franklin Lakes, NJ, USA) followed by a 1-h incubation at room temperature in the second secondary biotinylated goat anti-mouse (1:600, Vector laboratories #BA-9200, Burlingame, CA, USA), followed by streptavidin-594 (1:2000, ThermoFisher Scientific #S11227, Waltham, MA, USA).

### Microscopy

Immunostained brain sections and cells were observed with an inverted light/epifluorescence microscope (Olympus IX50; Olympus Corporation, Tokyo, Japan), equipped with a digital camera (Olympus XC 30, Olympus Corporation, Tokyo, Japan) and cellSens Software (Olympus Corporation, Tokyo, Japan). Captured images were adjusted for brightness and contrast with Adobe Photoshop (Adobe system, San Jose, CA, USA) software.

### Statistical analysis

Comparisons between two goups were performed using the parametric Student’s t-test or the non parametric Mann Whitney test. The comparison between more than two groups, where n was too small for D'Agostino-Pearson test of normality, was made using Kruskal–Wallis one-way analysis of variance with Dunn's multiple comparison test. For comparison of normally distributed data from more than two groups, 1-way or 2-way ANOVA followed by Bonferroni post hoc test were employed. Data are presented as mean ± s.e.m. P<0.1 was considered as a trend, and P<0.05 as significant. All data were analyzed using GraphPad Prism (GraphPad Software, San Diego, CA, USA).

## Results

### Treatment with MS-275 and resveratrol reduced infarct size and neurological deficits two days after pMCAO

**Fig. 1:**
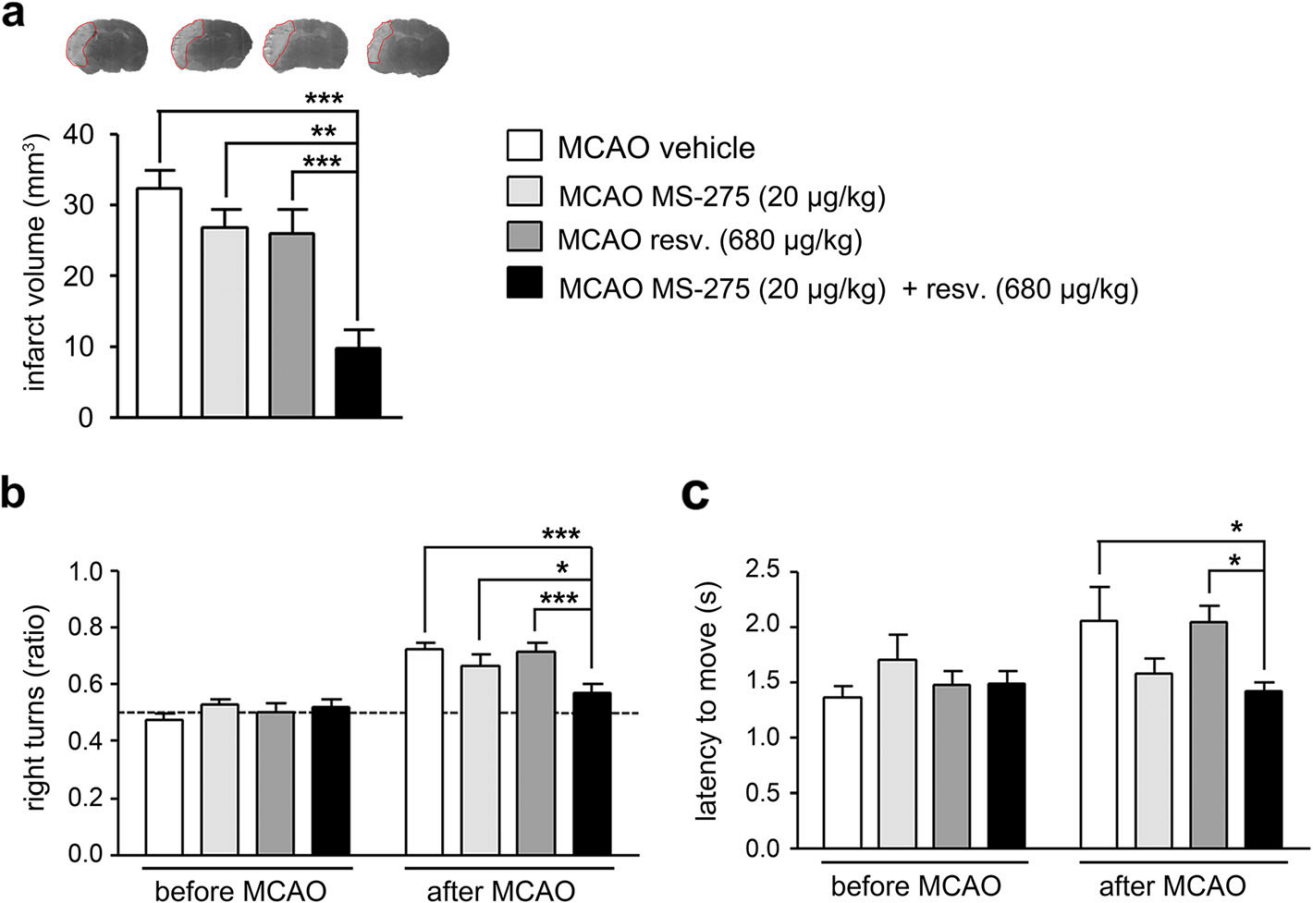
The combination of MS-275 and resveratrol is neuroprotective two days after pMCAO. (**a**) Treatment with MS-275 (20 μg/kg) and resveratrol (680 μg/kg) reduced the infarct volume, determined 2 days after pMCAO. Silver-stained coronal brain sections showing infarcts in light grey are depicted on top of the panel. ANOVA, **P<0.01 ***P<0.001 (Bonferroni post hoc test). Values are means ± s.e.m. (*n* = 9-11 from five independent experiments). (**b**) In the corner test, vehicle-, MS-275- and resveratrol-treated mice showed a preference to turn to the right side 2 days after pMCAO. Treatment with MS-275 and resveratrol reduced this preference for right turns. The dashed line indicates the expected behaviour without a side preference. Two-way repeated-measures ANOVA, *P<0.05, ***P<0.001 (Bonferroni post hoc test). Values are means ± s.e.m. (*n* = 11-13 from five independent experiments). (**c**) Treatment with MS-275 and resveratrol improved the latency to move that was increased 2 days after pMCAO. Two-way repeated-measures ANOVA, *P<0.05 (Bonferroni post hoc test). Values are means ± s.e.m. (*n* = 11-13 from five independent experiments).

The mouse model of permanent ischemia was accomplished by electrocoagulating the MCA. Treatments were administered i.p. immediately after the end of the procedure. To determine the synergistic protective effect of MS-275 plus resveratrol, the two drugs were injected individually (MS-275, 20 μg/kg; resveratrol, 680 μg/kg), or in combination (MS-275 20 μg/kg + resveratrol 680 μg/kg). We previoulsy showed that these doses were able to sinergistically reduce infarct volume and neurological deficits in mice subjected to tMCAO [15]. Experiments to assess the outcome of experimental stroke were conducted 2 days after pMCAO. After the permanent ischemia, infarcts were significantly smaller in mice treated with the combination of MS-275 (20 μg/kg) and resveratrol (680 μg/kg) than in vehicle- and single drug-treated animals (Fig. 1a). In parallel, only the treatment with the drug combination improved the pMCAO-induced neurological deficits as shown by the corner test. In 12 trials, mice tended to turn more often to the contralateral, that is right, than to the ipsilateral side after pMCAO. The combination of MS-275 and resveratrol normalized this preference for the right side observed in vehicle-, MS-275- and resveratrol-treated groups (Fig. 1b). Additionally, after pMCAO, the latency-to move one body length was prolonged. Only the treatment with the combination of MS-275 and resveratrol significantly improved this parameter (Fig. 1c).

### Treatment with MS-275 and resveratrol reduced the inflammatory response after pMCAO

**Fig. 2:**
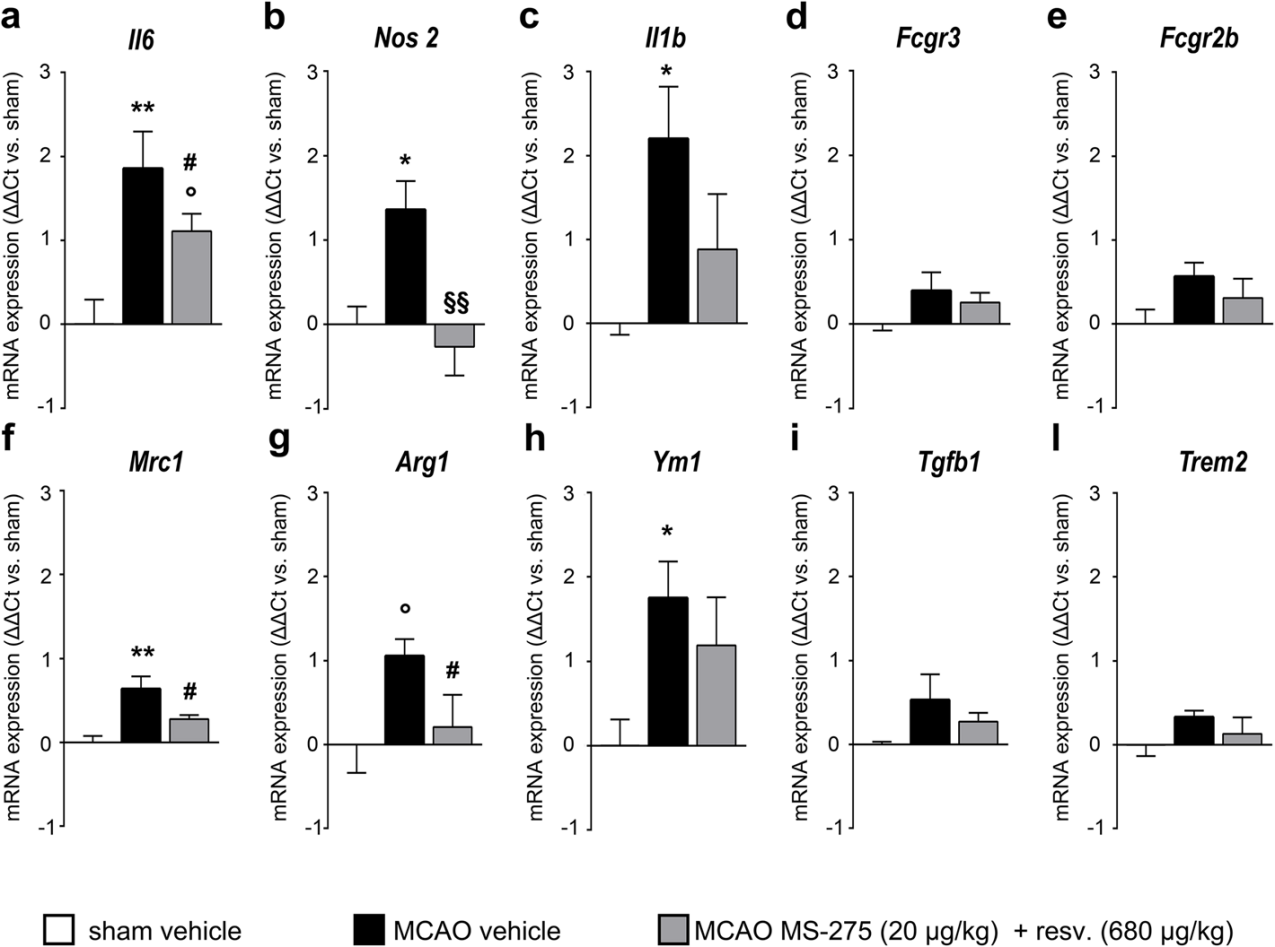
Inflammatory and microglia/macrophage mRNA expression profile in the ipsilateral hemisphere 1 day after pMCAO. (**a-l**) Quantification of *Il6, Nos2, Il1b, Fcgr3, Fcgr2b, Mrc1, Arg1, Ym1, Tgfb1 and Trem2* by qRT-PCR in the ipsilateral hemisphere 1 day after pMCAO. Following pMCAO, the expression of (**a**) *Il6*, (**b**) *Nos2*, (**c**) *Il1b*, (**f**) *Mrc1* and (**h**) *Ym1* was higher, and (**g**) *Arg1* showed an upward trend, if compared to the sham vehicle group. Treatment with MS-275 and resveratrol promoted a mild reduction of the expression of (**a**) *Il6,* (**f**) *Mrc1* and (**g**) *Arg1*, and prevented the increase of (**b**) *Nos2*, (**c**) *Il1b* and (**h**) *Ym1* transcript levels. One-way ANOVA followed by Holm-Šídák test, °trend, *p<0.05, **p<0.01 vs. sham vehicle; ^#^trend, ^§§^*p*<0.001 vs. MCAO vehicle. Data (means ± s.e.m., *n =* 6) are expressed as fold changes over values obtained in ipsilateral hemisphere from sham operated mice.

In order to study the effect of MS-275 and resveratrol on the early inflammatory status following pMCAO, ipsilateral hemispheres were analyzed for mRNA expression of inflammatory markers 1 day after a single post-insult administration of the drugs. The transcripts of several important players of the inflammatory and microglial/macrophage response in cerebral ischemia were analysed (Fig. 2). IL-6 (*Il6*) and IL-1β (*Il1b*) are two crucial pro-inflammatory cytokines in experimental and human stroke [36]. CD16 (*Fcgr3*) and CD32 (*Fcgr2b*) are surface receptors for immunocomplexes associated with activated microglia/macrophages where *Fcgr2b* acts by driving the polarization of microglia/macrophages toward an anti-inflammatory phenotype [37]. iNOS (*Nos2*), via the production of NO, is an important cytotoxic enzyme in cerebral ischemia [38]. MRC1 (*Mrc1*), Ym1 (*Ym1*) and Arg1 (*Arg1*) are markers of alternatively activated microglia and macrophages [39]. TGF-β1 (*Tgfb1*) is one of the main contributors to the anti-inflammatory environment after ischemic brain damage [38]. TREM2 (*Trem2*) is a receptor associated with the phagocytic activity of microglia [40]. qRT-PCR analysis of ipsilateral hemispheres showed a general increase of inflammation and the activation of microglia/macrophages 1 day after pMCAO. Permanent MCAO significantly induced the transcription of *Il6*, *Nos2, Il1b, Mrc1* and *Ym1*(Fig. 2a-c, f, h), and caused an upward trend in the *Arg1* expression (Fig. 2g). The expression of *Fcgr3, Fcgr2b, Tgfb1 and Trem2* remained unchanged (Fig. 2d, e, i, l). When compared to vehicle, the treatment with MS-275 (20 μg/kg) and resveratrol (680 μg/kg) globally prevented the inflammatory response by reducing the expression *of Nos2, Il1b, Il6* (Fig. 2a-c) as well as the microglia/macrophage alternative activation markers *Mrc1* and *Ym1* (Fig. 2f, g).

When analized in mice 7 days after the MCAO, either pro- or anti-inflammatory transcripts appeared elevated in comparison with the sham condition (additional file 1). With the exception of reduced *Il6* transcription, the expression of pro-inflammatory *Nos2* and *Fcgr3* increased, while *Il1b* and *Fcgr2b* maintained a trend of increase. In line with a progressive elevation of the anti-inflammatory/neuroprotective response of microglia and infiltrating macrophages in the post stroke period [27, 32, 41, 42], at 7 days *Mrc1, Tgfb1* and *Trem2* transcripts also significantly increased*.* At this time, the inhibitory effect of MS-275 and resveratrol was no longer evident on pro-inflammatory signals. Though the drug combination further boosted the expression of markers for microglia/macrophage alternative activation state by elevating transcription of *Fgcr2b* together with *Ym1* and *Arg1* (additional file 1).

### Treatment with MS-275 and resveratrol decreased the binding of RelA to *Nos2* promoter one day after pMCAO

**Fig. 3:**
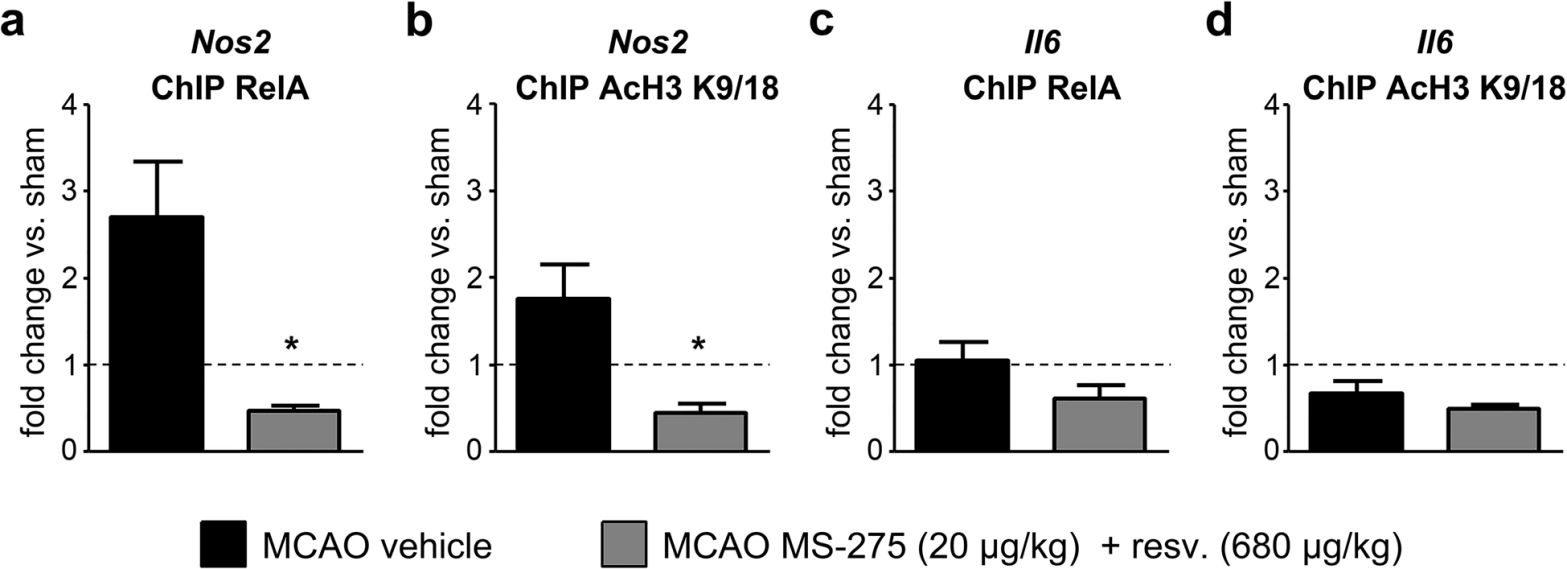
ChIP analysis of RelA binding and histone H3 acetylation (K9/18) at *Nos2* and *Il6* promoters. ChIP analysis of RelA binding and histone H3 acetylation (K9/18) at the (**a**, **b**) *Nos2* and (**c**, **d**) *Il6* promoters. Treatment with MS-275 and resveratrol reduced the pMCAO-induced increase in both (**a**) RelA binding and (**b**) the acetylation of H3 histone at the *Nos2* promoter. Permanent MCAO did not increase (**c**) RelA binding and (**d**) the acetylation of H3 histone at the *Il6* promoter. Data (means ± s.e.m., *n* = 3) are expressed as fold changes over values obtained in ipsilateral hemisphere from sham operated mice (dashed line). t-test, *p<0.05 versus pMCAO-vehicle.

*Nos2* and *Il6,* two pro-inflammatory genes modified by MS-275 and resveratrol treatment, are target of NF-ĸB-mediated transcriptional regulation [43]. We analysed the interactions between RelA and *Nos2* or *Il6* promoters in the cortex of mice 1 day after pMCAO, as well as the capability of the treatment with MS-275 (20μg/kg) and resveratrol (680μg/kg) to modulate that interaction. After chromatin immunoprecipitation with antibodies for Ac-H3(K9/18) and RelA, the relative level of *Nos2* and *Il6* promoters associated with acetylated H3 histone and bound RelA was determined by real-time qRT-PCR analysis, using primers that amplified the sequence of the *Il6* and *Nos2* promoters that include the κB binding sites. One day after pMCAO, both RelA binding and H3 acetylation at the *Nos2* promoter increased (Fig. 3a, b), whilst both remained unchanged at the *Il6* promoter (Fig. 3c, d). It suggests that the acetylation of H3 histone close to κB binding site was driven by the RelA binding to the promoter. When compared to vehicle, the treatment with MS-275 and resveratrol decreased the binding of RelA and the acetylation of H3 histone at the *Nos2* promoter, but only slightly decreased those at the *Il6* promoter (Fig. 3a-d).

### Treatment with MS-275 and resveratrol reduced leukocytes infiltration and microglia/macrophage inflammatory profile

**Fig. 4:**
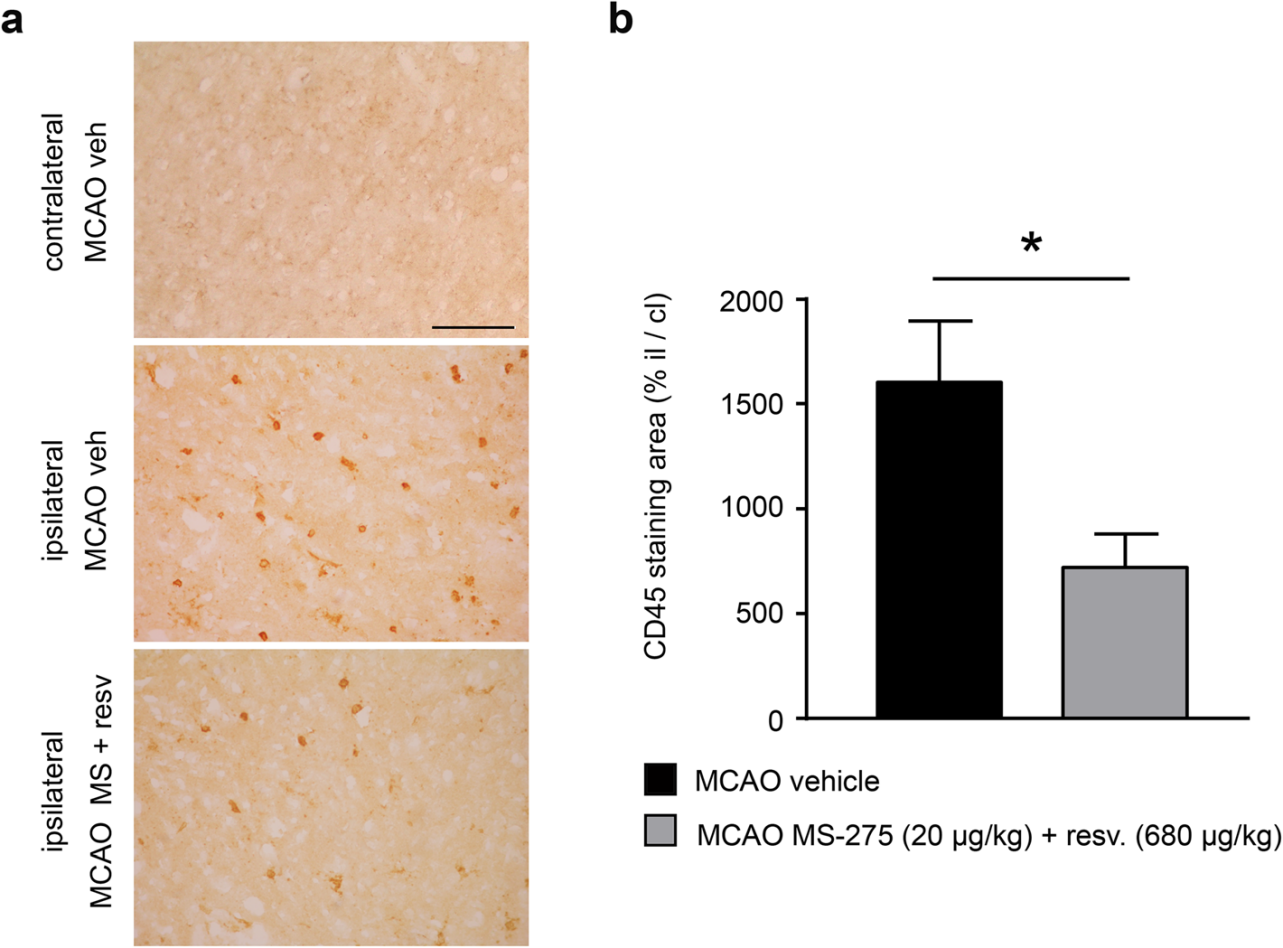
The combination of MS-275 and resveratrol reduces immunoreactivity for CD45 1 day after pMCAO. (**a**) Representative images of CD45 immunoreactivity in the peri-infarct area of brain sections obtained from the contralateral and ipsilateral hemispheres of MCAO vehicle, and from the ipsilateral hemisphere of MCAO MS-275 (20 μg/kg) + resveratrol (680 μg/kg) mice. Scale bar: 120 μm. (**b**) Quantification of CD45 immunostaining displays an effect of MS-275 and resveratrol treatment in reducing leukocytes infiltration. Data (means ± s.e.m. of 36-48 frames/mouse, *n =* 3) are expressed as CD45 positive area in the ipsilateral hemisphere/ CD45 positive area in the contralateral hemisphere. Mann Whitney test, *p < 0.05.

Analysis of infiltrating leukocytes was carried out by evaluating CD45 immunoreactivity (Fig. 4). One day after MCAO, the CD45-positive cells significantly increased in the ipsilateral penumbra area when compared to the contralateral hemisphere (Fig. 4a). MS-275 and resveratrol reduced the leukocyte infiltration, in line with the anti-inflammatory effect induced by the treatment (Fig. 4a, b). This positive effect was lost at 7 days where the higher leukocyte infiltration in ipsilateral ischemic emisphere appeared elevated in mice treated with MS-275 and resveratrol as in vehicle-treated MCAO mice (additional file 2).

**Fig. 5:**
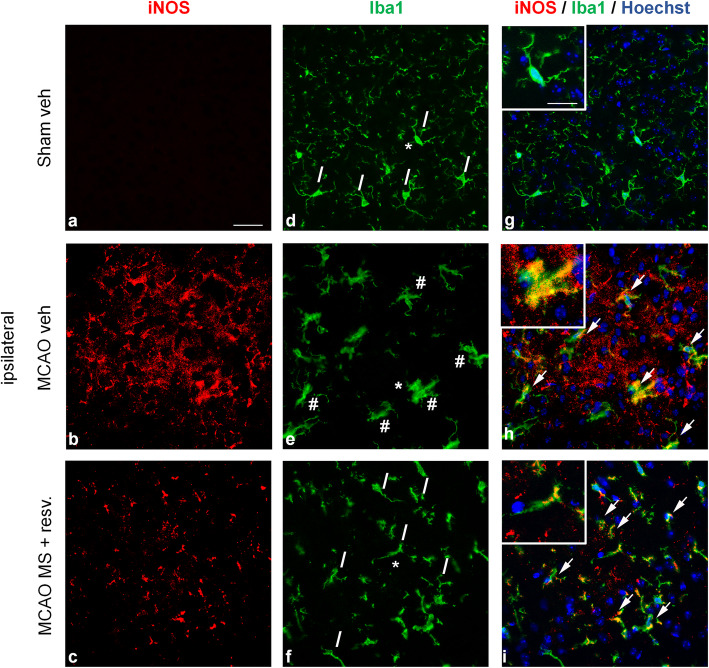
The combination of MS-275 and resveratrol reduces immunoreactivity for iNOS and Iba1 1day after pMCAO. (**a-i**) Representative images of iNOS (red) and Iba1 (green) immunofluorescence with Hoechst (blue) staining in the peri-infarct area of brain sections obtained from the ipsilateral hemispheres of (**a, d, g**) sham vehicle, (**b, e, h**) MCAO vehicle, and (**c, f, i**) MCAO MS-275 (20 μg/kg) + resveratrol (680 μg/kg) mice. # = bushy Iba1+ cells, / = ramified Iba1+ cells. Arrows represent sites of co-reactivity to iNOS and Iba1. Asterisks denote area shown in insets in higher magnification. Images are representative of 3 animals per group. Scale bars: in **a** = 50 μm for (**a**-**i**); in the inset in **g** = 20 μm for the insets in **g**, **h** and **i**.

**Fig. 6:**
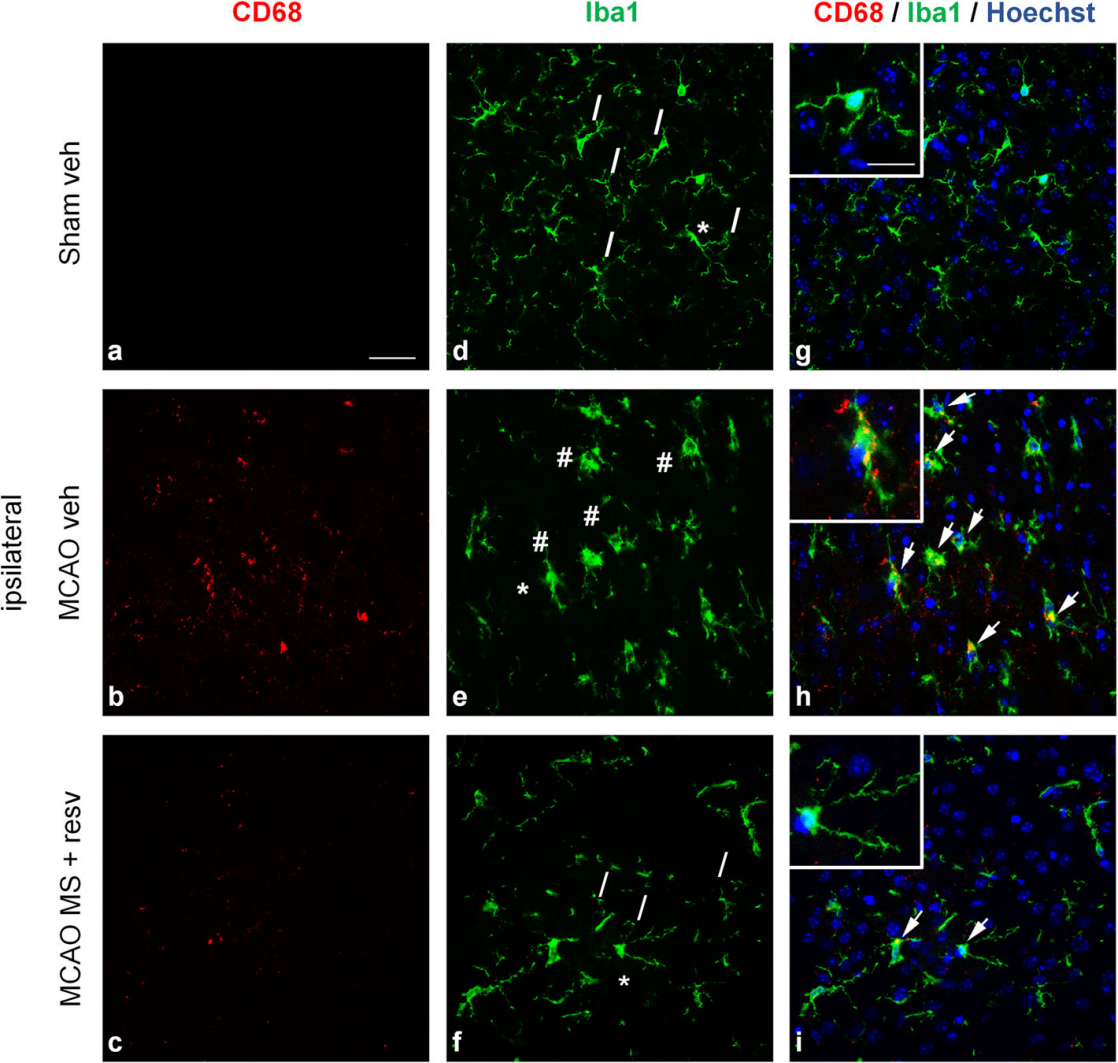
The combination of MS-275 and resveratrol reduces immunoreactivity for CD68 and Iba1 1 day after pMCAO. (**a-i**) Representative images of CD68 (red) and Iba1 (green) immunofluorescence with Hoechst (blue) staining in the peri-infarct area of brain sections obtained from the ipsilateral hemispheres of (**a, d, g**) sham vehicle, (**b, e, h**) MCAO vehicle, and (**c, f, i**) MCAO MS-275 (20 μg/kg) + resveratrol (680 μg/kg) mice. # = bushy Iba1+ cells, / = ramified Iba1+ cells. Arrows represent sites of co-reactivity to CD68 and Iba1. Asterisks denote area shown in insets in higher magnification. Images are representative of 3 animals per group. Scale bars: in **a** = 50 μm for (**a**-**i**); in the inset in **g** = 20 μm for the insets in **g**, **h** and **i**.

When analysing tissue from ipsilateral hemispheres, the results of real-time qRT-PCR for inflammatory genes cannot distinguish changes in single cell types. After the surprising finding that MS-275 and resveratrol treatment decreased pro- and anti-inflammatory transcripts 1 day after pMCAO, we evaluated the pro-inflammatory activation state of microglia/macrophages in the ischemic brains using their common markers Iba1 and iNOS. One day after pMCAO, the morphology of Iba1+ cells was analysed in the peri-infarct area of the ipsilateral hemisphere. Permanent ischemia induced the activation of microglia/macrophages that acquired a bushier, less ramified morphology (Fig. 5e, Fig. 6e, additional file 3e, cells marked with #). Conversely, Iba1+ cells from MS-275 and resveratrol-treated mice recovered a more ramified shape, similar to that displayed by the sham group of mice (Fig. 5f, Fig. 6f, additional file 3f, cells marked with /). Immunofluorescence for the pro-inflammatory marker iNOS was increased 1 day after pMCAO, compared with sham animals (Fig. 5b).

The treatment with MS-275 and resveratrol decreased the reactivity to iNOS (Fig. 5c). Not all iNOS+ cells were positive for Iba1, which is consistent with the fact that other cells, such as astrocytes and neurons, express iNOS and can contribute to the observed increased transcription [44] (Fig. 5g-i).

CD68 is a lysosomal glycoprotein associated with an increased phagocytic activity [45]. In control conditions, microglia do not express CD68, while after brain ischemia, activated microglia have been found positive for CD68 [46, 47]. One day after pMCAO, the CD68 immunoreactivity increased in cortical section, and the treatment with MS-275 and resveratrol lowered the CD68 signal (Fig. 6a-c). Some of the CD68+ cells were also Iba1+, but others negative (Fig. 6g-i). The latter population, CD68+/Iba1- cells, could very well be neutrophils known to infiltrate the brain 24h after pMCAO [27, 47, 48], as they are Iba1- cells endowed with phagocytic capacity and positivity to CD68 [47]. The immunoreactivity to MRC1 remained unchanged in all three experimental groups: sham, vehicle, and treated (additional file 3a-i). No detectable immunoreactivity for iNOS, CD68, or MRC1 was evident in the contralateral hemisphere of all animal groups.

Taken together, these data showed that the treatment with the combination of MS-275 and resveratrol diminished the pro-inflammatory activation state of microglia/macrophages. It was associated with a reduced reactivity to iNOS and CD68, as well as with the recovery of a “resting-like” ramified shape.

### Treatment with MS-275 and resveratrol prevented toxicity and expression of inflammation markers in mixed glial cultures incubated with conditioned medium from neurons subjected to OGD

**Fig. 7:**
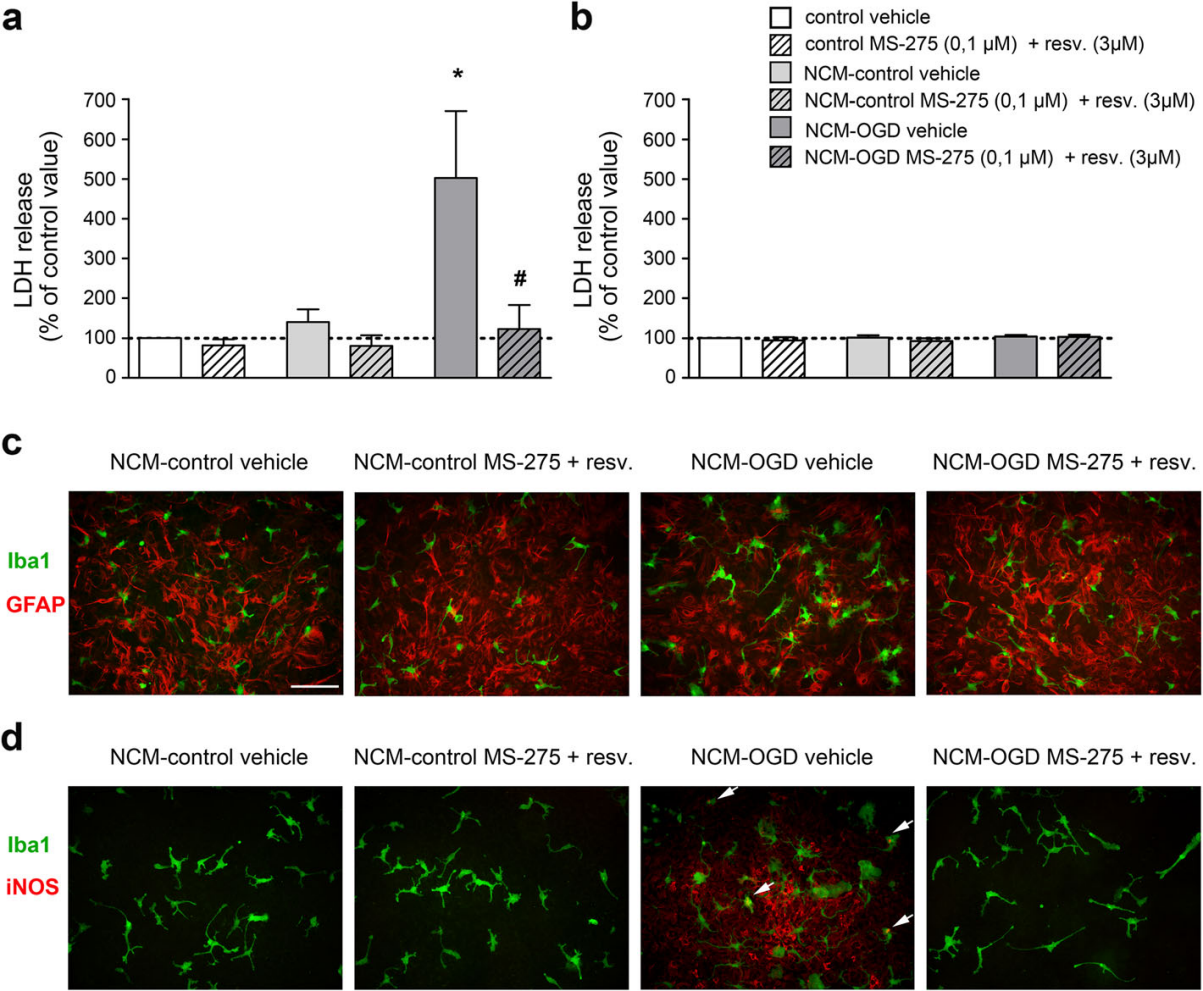
The combination of MS-275 and resveratrol reduces microglia toxicity and activation promoted by NCM. (**a**) LDH release from mixed glial cultures exposed to NCM. Mixed glial cultures were incubated overnight with NCM obtained from neurons subjected to OGD (NCM-OGD) or from control neurons (NCM-control). NCM-OGD induced significant toxicity in glial cells. The association of MS-275 (0.1 μM) and resveratrol (3 μM) significantly reduced the cellular damage mediated by NCM-OGD. Each value is expressed as the mean ± s.e.m. of 5 different replicate experiments, each performed in triplicate. *p<0.05, one-way ANOVA followed by Tukey multiple comparison test. (**b**) LDH release from pure astrocyte cultures exposed to NCM. Primary astrocytes were incubated overnight with NCM-OGD or NCM-control. The exposure to NCM did not increase the release of LDH from astrocytes. Each value is expressed as the mean ± s.e.m. of 3 different replicate experiments, each performed in triplicate. p>0.05, one-way ANOVA followed by Tukey multiple comparison test. (**c, d**) Representative images of (**c**) Iba1 (green) and GFAP (red) and (**d**) Iba1 (green) and iNOS (red) immunofluorescence staining on mixed glial cells incubated with NCM-OGD or NCM-control. Cultures incubated overnight with NCM-OGD displayed increased immunofluorescence for iNOS and morphological signs of activation in GFAP+ astrocytes and Iba1+ microglial cells. In (**d**), arrows in NCM-OGD image represent sites of co-reactivity to iNOS and Iba1. Treatment with MS-275 (0.1 μM) and resveratrol (3 μM) reduced reactivity to iNOS, and promoted recovery of the signs of glial cells activation in Iba1+ and GFAP+ cells. Images are representative of 3 experiments per group. Scale bar: in **c** = 50 μm for **c** and **d**.

In order to study the direct effect of the combination of MS-275 and resveratrol on microglial activation secondary to neuronal damage, we incubated primary mixed glial cells, or pure astrocytes, overnight with conditioned medium, obtained from neurons exposed to OGD (NCM-OGD), as well as from control neurons (NCM-control). The release of LDH, a marker of cellular injury, was significantly higher in mixed glial cells exposed to NCM-OGD when compared to control cultures (Fig. 7a). To identify possible direct effects of the combination of MS-275 and resveratrol in quenching microglial activation, we tested the treatment condition already shown to elicit synergistic neuroprotection in cortical neurons exposed to OGD [15]. The combination of MS-275 (0.1 μM) and resveratrol (3 μM), added concomitantly with the exposure to NCM-OGD, significantly decreased the LDH release (Fig. 7a).

By contrast, overnight incubation of pure astrocytes with NCM-OGD did not induce significant release of LDH (Fig. 7b), suggesting that microglia present in mixed glial cultures are crucial sensors of danger-associated molecular patterns (DAMPs) released by damaged neurons.

After incubation with NCM-OGD in mixed glial cultures, both Iba1+ microglia and GFAP+ astrocytes displayed larger bodies and thicker processes, that are morphological signs of activation (Fig. 7c). Moreover, mainly in astrocytes, but also in microglia, iNOS immunoreactivity greatly increased after exposure to NCM-OGD (Fig. 7d). The treatment with MS-275 and resveratrol reduced both the signs of glial cells activation (Fig. 7c) and the iNOS immunoreativity (Fig. 7d).

## Discussion

The first aim of our work was to investigate the capability of MS-275 and resveratrol to elicit a synergistic neuroprotection when administrated in combination in a mouse model of pMCAO. Analysis of the infarct volume 2 days after pMCAO showed that a single post-insult treatment with the combination of MS-275 (20 μg/kg) and resveratrol (680 μg/kg) was neuroprotective when compared to vehicle, or administration of individual drugs administered separately. These data were accompanied by the improvement in the corner and latency-to-move trials, two established tests to assess sensorimotor function in pMCAO-induced neurological deficits [30]. These data, supporting the valuable effect of MS-275 and resveratrol in the acute treatment of ischemic stroke [15], are compatible with evidence of protective activity elicited by HDACi alone, though at a 100-fold higher dose, in models of brain ischemia [17, 18, 21].

In recent years, a body of evidence has shown that pMCAO induces a dual-phase activation of microglia/macrophages that evolve with the time: microglia/macrophage expressing markers for either alternative or classical activation state are induced at 24 hours and further increase at later stages [32]. We investigated the effect of the synergistic combination of MS-275 and resveratrol on the inflammatory transcription 1 day after pMCAO. At that time, both pro- (*Il6*, *Nos2* and *Il1b*) and anti-inflammatory markers (*Mrc1* and *Ym1*) appeared increased. This is in agreement with published data describing the inflammatory response observed 15 hours after the onset of pMCAO [49]. MS-275 and resveratrol-treated animals showed a lesser increase of pro-inflammatory markers, particularly a reduced transcription of *Nos2*, in agreement with data showing that HDACi, such as valproic acid and sodium butyrate, exhibit anti-inflammatory effects in pMCAO [50]. In our case, however, treatment with MS-275 and resveratrol also decreased the rise of anti-inflammatory markers, suggesting a general inhibitory effect on the inflammatory reaction, including the microglia/macrophage activation [49]. The anti-inflammatory effect of MS-275 and resveratrol waned at a longer post-ischemic time. At 7 days, the treatment did not reduce the sustained increase of pro-inflammatory *Nos2* and *Fcgr3,* as well as the increase of anti-inflammatory *Mrc1, Tgfb1* and *Trem2* transcripts. Yet, the treatment enhanced the expression of *Fgcr2b* and alternative activation markers of microglia/macrophages, *Ym1* and *Arg1.* In view of the opposite regulation elicited by the *Fgcr* immunocomplex receptors on immune cells, *Fcgr3* stimulatory and *Fcgr2b* inibitory of the pro-inflammatory state [37], it can be assumed that MS-275 and resveratrol enhanced the polarization of immune cells, including microglia/macrophages, toward an anti-inflammatory mode at a later post-ischemic phase. A condition to which peripheral immune cells as monocyte-derived macrophages highly contribute [41].

In line with the drug-induced reduction of inflammatory profile and infarct volume, the analysis of the area of CD45-positive cells in treated mice showed a reduced leukocytes infiltration 1 day after MCAO. No change was evident in the enhanced infiltration at 7 days after the ischemic insult in treated mice.

This result suggests that, although ineffective in reducing the upregulation of inflammatory gene transcripts and leukocytes brain invasion at this longer interval (7 days), the drug single administration was effective in driving later infiltrating monocytes toward a protective, anti-inflammatory profile. An evidence that makes compelling the study of repeated administrations of the drugs after pMCAO.

To assess whether the results observed at 24 hours were due to an epigenetic effect, we performed a ChIP analysis at the *Nos2* promoter, a NF-ĸB target gene [43] whose transcription was inhibited by the treatment. The assay revealed a strong decrease in RelA binding and H3 acetylation at the *Nos2* promoter in ipsilateral hemispheres from mice treated with MS-275 and resveratrol after the insult, compared to vehicle-treated animals. Another NF-ĸB target gene that was increased after pMCAO was *Il6*, but the ChIP results showed that RelA binding to its promoter did not increase after the insult, leading us to conclude that, at the tested time point, NF-ĸB is dispensable for *Il6* expression. This in line with evidence that the transcription of *Il6* in brain ischemia is also regulated by CREB [51], C/EBPβ [52], and AP-1 [53], all transcription factors shown to cooperate with NF-ĸB in controlling the *Il6* transcript levels [54].

These results led us to hypothesize that microglia could be less activated. Although our experimental design did not allow us to distinguish between microglia and peripheral macrophages, in the peri-infarct area most of the Iba1+ cells are resident microglia 1 day after permanent ischemia [55]. With the aim of assessing the effect of the drug combination on the activation state of microglia/macrophages, we performed a double immunofluorescence labeling of Iba1 with iNOS and CD68, respectively a pro-inflammatory and a phagocytosis markers. Our examination revealed Iba1+ cells on the peri-infarct area with mostly bushy/hypertrophic and ramified morphology, whereas round Iba1+ cells were rarely seen. The presence of thinly ramified Iba1+ cells, morphologically similar to those present in sham mice, was evident in the peri-infarct area of drug-treated animals, compared to vehicle-treated. This suggests that the administration of MS-275 and resveratrol decreases the activation of microglia/macrophages [56].

The combination of MS-275 and resveratrol reduced the immunoreactivity to iNOS, though this reduction is mainly seen in Iba1- cells. Other cell types, such as astrocytes, express this marker [44]. Iba1+/iNOS+ cells presented ramified and bushy morphologies, in agreement with recent published data [42].

Acute treatment of ischemic mice resulted in lowered immunoreactivity for CD68+ phagocytic cells. Phagocytosis in cerebral ischemia is a complex phenomenon and is not clearly linked to a specific pro-/anti-inflammatory state [57], or beneficial/deleterious effects [58]. Alike iNOS, CD68 was detected in Iba1- cells, which could be infiltrating phagocytic CD68+/Iba1- neutrophils [47], while CD68+/Iba1+ were ramified and bushy cells, as was previously shown [42]. We found that, at 1 day post-pMCAO, the immunoreactivity for MRC1 did not change between sham, vehicle- or drug-treated animals. This seems in agreement with recent published data, at 1 day after pMCAO, showing that co-positivity for MRC1 and Iba1 is detectable exclusively in round, ameboid cells [42]. It should be noted that, although significant, the increase of the mRNA of MRC1 at 1 day is minimal. Therefore, this immunofluorescence finding is in line with the low-grade expression of MRC1 detected after pMCAO.

Our results show that treatment with MS-275 and resveratrol decreases the pro-inflammatory activation of Iba1+ microglia/macrophages in the peri-infarct area, by reducing their immunoreactivity to iNOS and CD68.

The last step was to confirm whether the anti-inflammatory effect seen *in vivo* was neuron-mediated or if the treatment could elicit a direct anti-inflammatory effect on glial cells. With the intent of using a cellular model that could mirror some of the complexity of the post-ischemic environment and players, primary cultures of mixed glial cells and pure astrocytes were incubated overnight with MS-275 and resveratrol in a NCM from neurons exposed to OGD. The results showed that microglia are the first cells to sense and respond to damaged neurons, because only when they were present, in mixed glial cell culture, the exposure to NCM-OGD increased the release of LDH. The decrease in LDH release from mixed glial cells incubated with MS-275 and resveratrol demonstrates that the epigenetic drugs can directly target glial cells.

The increase in immunoreactivity for iNOS, seen after the incubation of mixed glial cell with NCM-OGD, is in line with RelA binding and histone H3 acetylation at the *Nos2* promoter observed in the pMCAO model. Noteworthy, iNOS activation was not observed exclusively in Iba1+ cells, but mostly in Iba1- cells, the astrocytes. We can assume that, although microglia are the first sensors of neuronal distress mediated by DAMPs, they rapidly communicate with other cells, disseminating the “message”.

## Conclusion

By combining these with previous findings [15], we can conclude that the aberrant acetylation of RelA, responsible for the pro-apoptotic transcription following noxious brain ischemia, is reverted by a single post-insult administration of MS-275 and resveratrol. The change in RelA acetylation hampers RelA interaction with the *Nos2* promoter and, by shifting the binding of transcription factor from the *Bim* to *Bcl-χ*_*L*_ *gene*, concomitantly reduces the pro-inflammatory transcription and promotes the anti-apoptotic one. MS-275 and resveratrol synergistically reduced brain damage and neurological deficits, showing to be more efficient than individual drugs used at 100-fold higher doses [15]. We can assume that the neuroprotective activity observed 2 days after pMCAO occurred through different, complementary mechanisms. The drugs induced neuron survival and, both directly and indirectly, reduced pro-inflammatory activation of microglia/macrophages. Along with the inhibition of the early inflammatory response, the treatment also limited the leukocytes infiltration thus quenching further the expression of pro-inflammatory genes. The beneficial effects of a single administration was not permanent, although it could drive the later infiltrating monocytes toward a protective anti-inflammatory phenotype.

Our data supports the use of MS-275 and resveratrol in treating acute ischemic stroke at a very low doses, with less risk of side effects. Future experiments testing repeated administration of these drugs during the subacute phase will unveil the possibility to maximize the functional recovery after stroke.

## Supplementary Information

**Additional file 1. **Inflammatory and microglia/macrophage mRNA expression profile in the ipsilateral hemisphere 7 days after pMCAO. (**a-l**) Quantification of *Il6, Nos2, Il1b, Fcgr3, Fcgr2b, Mrc1, Arg1, Ym1, Tgfb1* and *Trem2* by qRT-PCR in the ipsilateral hemisphere 7 days after pMCAO. Seven days since stroke induction, expression of (**g**) *Arg1* and (**h**) *Ym1* was not different from sham vehicle group. Transcript levels of (**b**) *Nos2*, (**d**) *Fcgr3*, (**f**) *Mrc1*, (**i**) *Tgfb1* and (**l**) *Trem2* were significantly higher when compared to sham vehicle group, while (**c**) *Il1b* and (**e**) *Fcgr2b* showed an upward trend. Expression of (**a**) *Il6* was significantly lower. Treatment with MS-275 and resveratrol did not increase the expression of (**a**) *Il6* , nor reduce the transcript levels of (**b**) *Nos2,* (**d**) *Fcgr3,* (**f**) *Mrc1,* (**i**) *Tgfb1* and (**l**) *Trem2*. Instead, the treatment enhanced the expression of (**e**) *Fcgr2b*, (**g**) *Arg1* and (**h**) *Ym1.* One-way ANOVA followed by Holm-Šídák test, °trend, *p<0.05, **p<0.01, ***p<0.001, ****p<0.0001 *vs.* sham vehicle; #trend, §§§*p*<0.001 *vs.* MCAO vehicle. Data (means ± s.e.m., *n* = 6) are expressed as fold changes over values obtained in ipsilateral hemispheres from sham operated mice.

**Additional file 2. **CD45 immunoreactivity in the peri infarct area 7 days after pMCAO. (**a**) Representative images of CD45 immunoreactivity in the peri-infarct area of brain sections obtained from the controlateral and ipsilateral hemispheres of MCAO vehicle, and from the ipsilateral hemisphere of MCAO MS-275 (20 μg/kg) + resveratrol (680 μg/kg) mice. (**b**) Quantification of CD45 immunostaining shows the absence of MS-275 and resveratrol treatment effect in reducing leukocytes infiltration. Data (means ± s.e.m. of 36-42 frames/mouse, *n* = 3) are expressed as CD45 positive area in the ipsilateral hemisphere/ CD45 positive area in the contralateral hemisphere. Scale bar: 120 μm. Mann Whitney test, p > 0.05.

**Additional file 3. **MRC1 and Iba1 expression in the peri infarct area 1 day after MCAO (**a-i**) Representative images of MRC1 (red) and Iba1 (green) immunofluorescence with Hoechst (blue) staining on brain sections obtained from the ipsilateral hemispheres of (**a, d, g**) sham vehicle, (**b, e, h**) MCAO vehicle, and (**c, f, i**) MCAO MS-275+resv. mice. # = bushy Iba1+ cells, / = ramified Iba1+ cells. Arrows represent sites of co-reactivity to MRC1 and Iba1. Asterisks denote area shown in insets in higher magnification. Images are representative of 3 animals per group. Scale bars: in **a** = 50 µm for (**a**-**i**); in the inset in **g** = 20 μm for the insets in **g**, **h** and **i**.

## Data Availability

The datasets and materials used and/or analysed during the current study are available from the corresponding author on reasonable request.

## Abbreviations

AMPK: AMP-activated kinase
ChIP: chromatin immunoprecipitation
DAMPs: danger associated molecular patterns
DMSO: dimethylsulfoxide
HATs: histone acetyltransferases
HDACi: histone deacetylase inhibitors
LDH: lactate dehydrogenase
NCM: neuron-derived conditioned medium
NF-κB: nuclear factor-kappa B
OGD: oxygen glucose deprivation
pMCAO: permanent middle cerebral artery occlusion
qRT-PCR: quantitative reverse transcription-polymerase chain reaction
rtPA: recombinant tissue plasminogen activator
tMCAO: transient middle cerebral artery occlusion
