# Supplementary figures and images for: Neuroprotective epi-drugs quench the inflammatory response and microglial/macrophage activation in a mouse model of permanent brain ischemia
Source: J Neuroinflammation. 2020 Nov 27;17:361. doi: 10.1186/s12974-020-02028-4 (PMC7694916; doi:10.1186/s12974-020-02028-4)

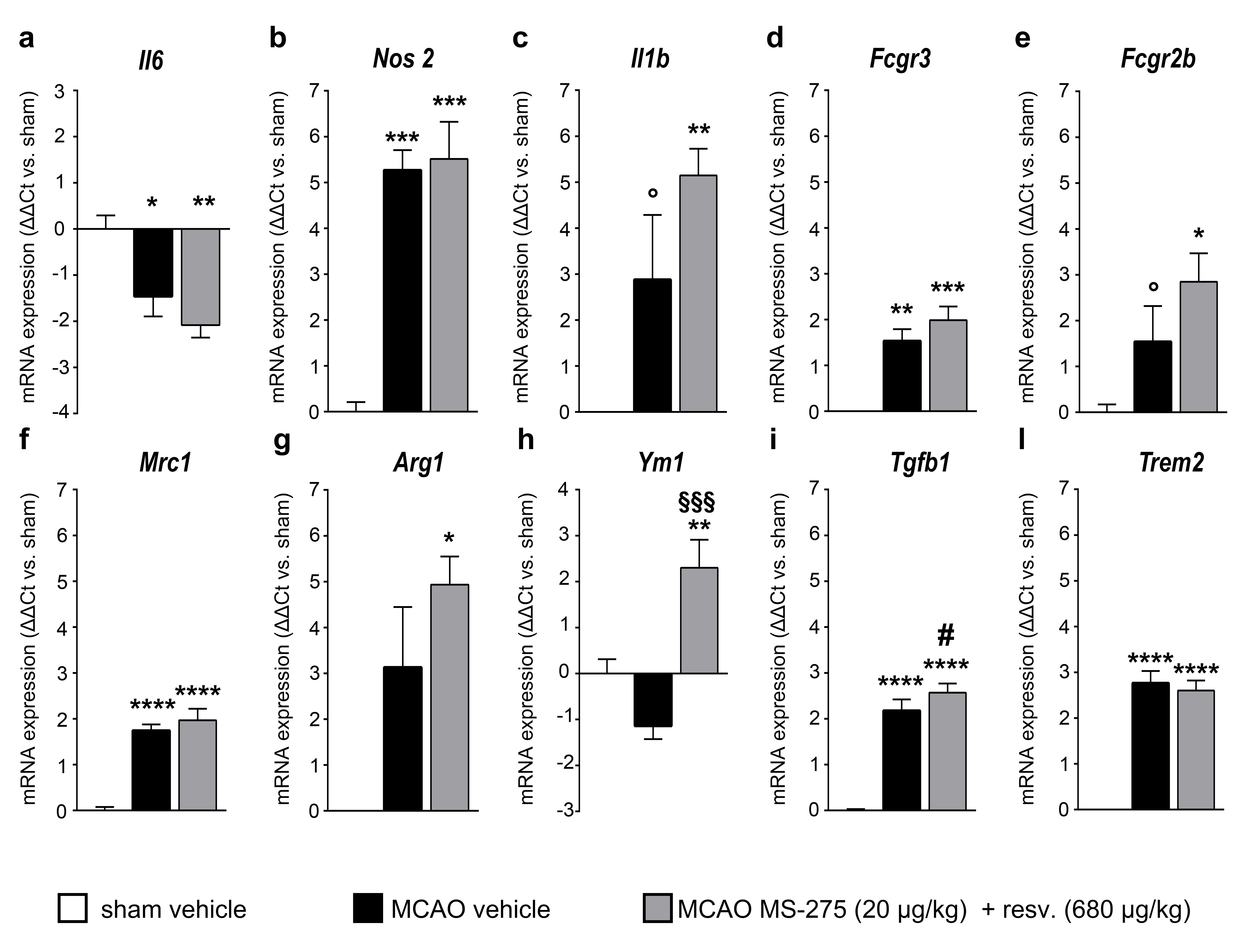

Supplement: Supplementary file 1 — Additional file 1. Inflammatory and microglia/macrophage mRNA expression profile in the ipsilateral hemisphere 7 days after pMCAO. (a-l) Quantification of Il6, Nos2, Il1b, Fcgr3, Fcgr2b, Mrc1, Arg1, Ym1, Tgfb1 and Trem2 by qRT-PCR in the ipsilateral hemisphere 7 days after pMCAO. Seven days since stroke induction, expression of (g) Arg1 and (h) Ym1 was not different from sham vehicle group. Transcript levels of (b) Nos2, (d) Fcgr3, (f) Mrc1, (i) Tgfb1 and (l) Trem2 were significantly higher when compared to sham vehicle group, while (c) Il1b and (e) Fcgr2b showed an upward trend. Expression of (a) Il6 was significantly lower. Treatment with MS-275 and resveratrol did not increase the expression of (a) Il6 , nor reduce the transcript levels of (b) Nos2, (d) Fcgr3, (f) Mrc1, (i) Tgfb1 and (l) Trem2. Instead, the treatment enhanced the expression of (e) Fcgr2b, (g) Arg1 and (h) Ym1. One-way ANOVA followed by Holm-Šídák test, °trend, *p<0.05, **p<0.01, ***p<0.001, ****p<0.0001 vs. sham vehicle; #trend, §§§p<0.001 vs. MCAO vehicle. Data (means ± s.e.m., n = 6) are expressed as fold changes over values obtained in ipsilateral hemispheres from sham operated mice. [file 12974_2020_2028_MOESM1_ESM.tif]

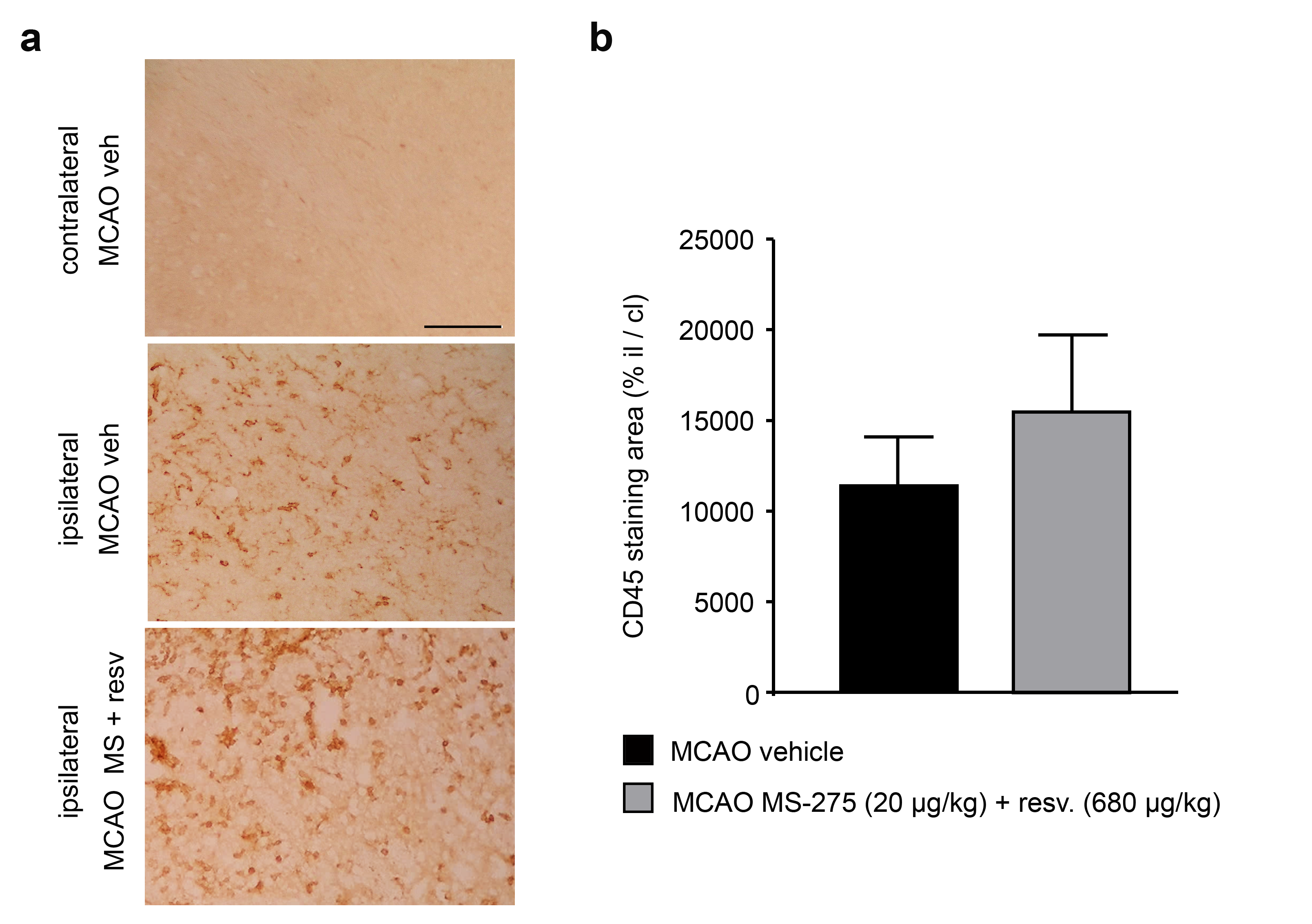

Supplement: Supplementary file 2 — Additional file 2. CD45 immunoreactivity in the peri infarct area 7 days after pMCAO. (a) Representative images of CD45 immunoreactivity in the peri-infarct area of brain sections obtained from the controlateral and ipsilateral hemispheres of MCAO vehicle, and from the ipsilateral hemisphere of MCAO MS-275 (20 μg/kg) + resveratrol (680 μg/kg) mice. (b) Quantification of CD45 immunostaining shows the absence of MS-275 and resveratrol treatment effect in reducing leukocytes infiltration. Data (means ± s.e.m. of 36-42 frames/mouse, n = 3) are expressed as CD45 positive area in the ipsilateral hemisphere/ CD45 positive area in the contralateral hemisphere. Scale bar: 120 μm. Mann Whitney test, p > 0.05. [file 12974_2020_2028_MOESM2_ESM.tif]

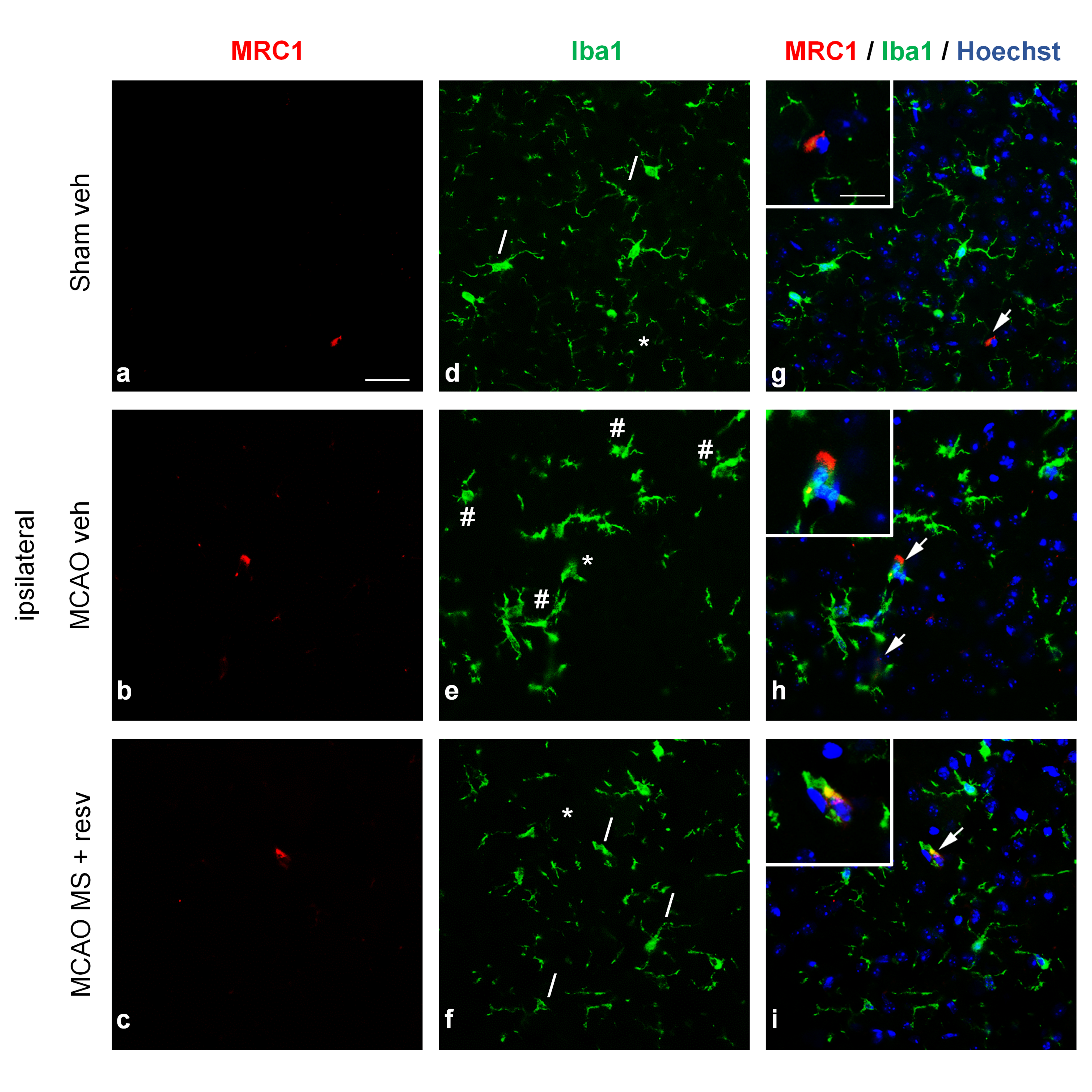

Supplement: Supplementary file 3 — Additional file 3. MRC1 and Iba1 expression in the peri infarct area 1 day after MCAO (a-i) Representative images of MRC1 (red) and Iba1 (green) immunofluorescence with Hoechst (blue) staining on brain sections obtained from the ipsilateral hemispheres of (a, d, g) sham vehicle, (b, e, h) MCAO vehicle, and (c, f, i) MCAO MS-275+resv. mice. # = bushy Iba1+ cells, / = ramified Iba1+ cells. Arrows represent sites of co-reactivity to MRC1 and Iba1. Asterisks denote area shown in insets in higher magnification. Images are representative of 3 animals per group. Scale bars: in a = 50 µm for (a-i); in the inset in g = 20 μm for the insets in g, h and i. [file 12974_2020_2028_MOESM3_ESM.tif]
